# Celecoxib induced bullous eruption confirmed by a patch test

**DOI:** 10.1186/2045-7022-4-S3-P81

**Published:** 2014-07-18

**Authors:** Karim Aouam, Najah Ben Fadhel, Nadia Ben Fredj, Amel Chaabane, Monia Youssef, Naceur A Boughattas

**Affiliations:** 1 Laboratory of Pharmacology, Department of Pharmacology, Tunisia; 2 Faculty of Medicine of Monastir, Department of Pharmacology, Tunisia; 3 University Hospital of Monastir, Department of Pharmacology, Tunisia

## Introduction

Celecoxib is a selective cyclooxygenase-2 (COX-2) inhibitor. It is frequently associated with several side effects such as urticaria, anaphylaxis, angio-oedema and erythema multiforme. However, bullous eruptions induced by celecoxib have been rarely described previously. We report an original case of Celecoxib-induced bullous eruption confirmed by a positive patch test.

## Case report

A 32-year-old woman was referred to the emergency department because she developed a skin eruption, pruritis and frisson which have occurred four hours after the intake of 200mg of celecoxib for inflammatory back pain. Physical examination revealed generalized urticaria which was thought to be induced by celecoxib. Therefore, she was prescribed corticosteroid therapy and antihistaminic drug and advised to avoid celecoxib. Three days later, she has returned to the emergency department because of skin eruption worsening. Physical examination showed bullous eruption predominantly in the proximal limbs and the buttock without mucosal involvement. The body temperature and blood pressure were normals. The laboratory findings didn't show any abnormality. When examining the patient medical history, we found that she has had developed, three years ago, a generalized skin eruption two days after celecoxib intake. Six weeks after total recovery of skin eruption, patch test to celecoxib (10% in petroleum) was performed, showing a positive reaction at 48h reading. In order to investigate a possible cross reactivity among sulfonamide drugs, we carried out a patch test to furosemide which was negative at 48h reading.

## Conclusion

We add to the medical literature a case of bullous eruption induced by celecoxib, confirmed by a positive skin test. Clinicians should be aware of such potentially severe side effect when prescribing this drug.

